# *Alistipes*-derived monomethyl branched-chain fatty acid iso-15:0 suppresses hepatocellular carcinoma progression by inhibiting Gβγ/PI3K/AKT signaling

**DOI:** 10.1186/s43556-026-00622-1

**Published:** 2026-10-14

**Authors:** Juan He, Yu Chen, Liwen Kuang, Yang Xiao, Jiongming Chen, Xudong Liu, Jiangang Zhang, Ziyi Li, Dongqin Zhou, Miaoyu Song, Yi Yi, Danyang Li, Haili Shang, Nan Zhang, Ran Ren, Yu Zhou, Juan Lei, Huakan Zhao, Yongsheng Li

**Affiliations:** 1 School of Medicine, Department of Medical Oncology of Chongqing University Cancer Hospital, Chongqing University, Chongqing, 400030 China; 2 Chongqing Key Laboratory for the Mechanism and Intervention of Cancer Metastasis, Chongqing, 400030 China; 3 Key Laboratory of Chongqing Education Commission of China for Cancer Immunometabolism Translational Research, Chongqing, 400030 China

**Keywords:** *Alistipes*, iso-15:0, HCC, Proliferation, Migration

## Abstract

The role of gut microbiota and their metabolites in the progression of hepatocellular carcinoma (HCC) has garnered increasing attention. Although *Alistipes*, a genus frequently reported as a beneficial bacterium, has been associated with antitumor effects and enhanced efficacy of cancer therapies, its specific functions and underlying mechanisms in HCC remain poorly defined. In this study, 16S rDNA sequencing revealed a marked decrease in the abundance of *Alistipes* in fecal samples from HCC patients compared to healthy controls, a finding that was also observed in HCC mouse models. Additionally, iso-15:0, an important fatty acid metabolite derived from *Alistipes*, was found to be significantly reduced in the feces of both HCC patients and HCC mouse models. Functional experiments demonstrated that iso-15:0 effectively suppresses the proliferation and migration of HCC cells in vitro and in vivo. Mechanistically, iso-15:0 inhibits the activation of the PI3K/AKT signaling pathway by interfering with G protein-coupled receptor (GPCR) downstream signaling, specifically through the Gβγ subunits. To further explore the potential for the clinical application of iso-15:0, we have developed a biomimetic nanodelivery system (3R/RGD/M1@iso-15:0 LNP) that markedly improves the delivery efficiency and therapeutic efficacy of iso-15:0 in vivo. Our findings elucidate for the first time that iso-15:0, derived from *Alistipes*, suppresses HCC progression by restricting the Gβγ–PI3K/AKT signaling axis, highlighting its potential as a novel therapeutic strategy for HCC.

**Supplementary Information:**

The online version contains supplementary material available at https://doi.org/10.1186/s43556-026-00622-1.

## Introduction

Hepatocellular carcinoma (HCC) continues to be a leading cause of cancer-related mortality worldwide, representing a significant global health burden [1]. Despite advances in therapeutic interventions such as surgery, radiotherapy, and targeted therapies, the long-term survival rates for HCC patients remain unsatisfactory, highlighting the urgent need for novel prevention and treatment strategies [2].

Accumulating evidence emphasizes the critical role of gut microbial dysbiosis in hepatocarcinogenesis [3]. Patients with advanced HCC exhibit an altered gut microbiota composition, characterized by an increase in pathobionts (e.g., *Enterococcus* spp. and *Catenibacterium* spp.) and a decrease in beneficial microbes (e.g., *Bifidobacterium* spp. and *Akkermansia* spp.) compared to healthy individuals [4, 5]. This dysbiosis is thought to facilitate HCC progression by enriching pathogenic bacteria and depleting protective commensals. Furthermore, microbial metabolites, including bile acids [6] and short-chain fatty acids [7], can translocate to the liver via the gut-liver axis, thereby initiating or exacerbating HCC development [8]. Conversely, certain probiotics and their metabolites have shown protective effects. Collectively, these findings suggest that elucidating the precise mechanisms through which specific gut microbes and their metabolites contribute to HCC pathogenesis could reveal novel avenues for prevention and treatment.

*Alistipes* is a relatively novel genus of Gram-negative bacteria within the *Bacteroidetes* phylum, primarily isolated from clinical samples [9]. However, its role in dysbiosis is dualistic, exhibiting both beneficial and detrimental effects across different disease contexts [9–11]. Notably, several studies have reported a significant association between *Alistipes* and liver diseases. Specifically, a decline in the abundance of *Alistipes* has been observed during the progression of liver fibrosis [12]. Consistent with this, the abundance of this genus is markedly reduced in fecal samples from patients with early-stage HCC, suggesting a potential protective role in hepatocarcinogenesis [4]. Furthermore, *Alistipes* species predominantly produce the branched-chain fatty acid metabolite iso-15:0 [13–16]. Although this branched-chain fatty acid has previously been studied primarily in contexts such as soybean fermentations or bovine milk [17, 18], its production by gut microbiota and its functional significance remains largely unexplored. Critically, the specific mechanism by which *Alistipes* and its metabolite iso-15:0 influence HCC progression remains unclear. Therefore, elucidating this relationship is imperative for the development of novel therapeutic strategies against HCC.

To investigate the role of *Alistipes*-derived metabolites in the malignant progression of HCC, we performed 16S rDNA and metagenomic sequencing on fecal samples obtained from HCC patients and mouse models. Through subsequent in vitro and in vivo functional studies, we systematically elucidated the mechanism by which iso-15:0, a key fatty acid metabolite produced by *Alistipes*, suppresses HCC progression. To further assess the therapeutic feasibility of iso-15:0, we employed a biomimetic nanodelivery strategy aimed at enhancing its efficiency in tumor delivery. Our study reveals for the first time that iso-15:0, derived from *Alistipes*, suppresses HCC progression by targeting the Gβγ/PI3K/AKT signaling axis. These findings provide substantial molecular evidence supporting the concept of the 'gut-liver axis' and establish a foundation for utilizing microbial metabolites as novel strategies and biomarkers for the precise prevention and treatment of HCC.

## Results

### Depletion of the *Alistipes* genus and its key fatty acid metabolite in HCC patients

**Fig. 1:**
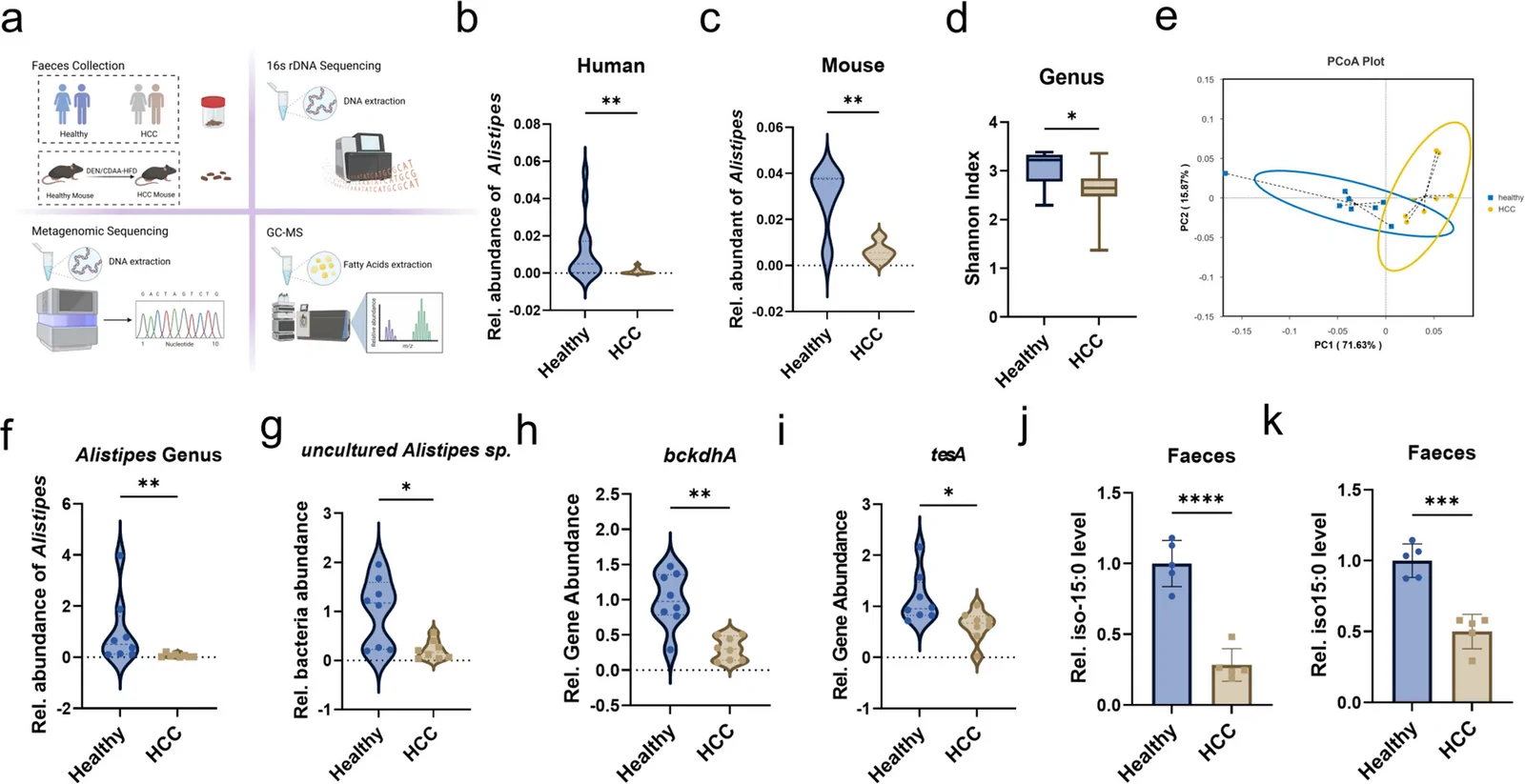
The genus *Alistipes* and its predominant fatty acid metabolites are depleted in HCC patients. **a** A schematic diagram illustrating the workflow for sample collection and detection. **b** The relative abundance of the *Alistipes* genus in fecal samples from HCC patients and healthy controls by 16S rDNA gene sequencing. **c** The abundance of *Alistipes* in fecal samples from DEN/CDAA-HFD-induced HCC mouse models by 16S rDNA gene sequencing (*n* = 5). **d** Genus-level α-diversity indices of fecal microbiota from HCC patients and healthy controls were calculated based on metagenomic sequencing data (*n* = 8). **e** The genus-level β-diversity of fecal microbiota between HCC patients and healthy controls was evaluated using metagenomic sequencing and visualized through PCoA(*n* = 8). **f** Metagenomic analysis of the relative abundance of the *Alistipes* genus in fecal samples from HCC patients and healthy controls (*n* = 8). **g** Species-level taxonomic profiling of the *Alistipes* genus based on metagenomic sequencing data (*n* = 8). **h**-**i** The relative gene abundance of the BckdhA and TesA genes in fecal samples from HCC patients compared to healthy controls was quantified using metagenomic sequencing (*n* = 8). **j** Fecal iso-15:0 levels in HCC patients and healthy controls were measured using GC–MS (*n* = 5). **k** Fecal iso-15:0 levels in DEN/CDAA-HFD-induced HCC mouse models were detected using GC–MS (n = 5). Data are presented as mean ± SD, with significance levels indicated as *p < 0.05, **p < 0.01, ***p < 0.001, ****p < 0.0001 and ns denotes not significant

To investigate the functional role of gut microbial dysbiosis in the malignant progression of HCC, we first performed 16S rDNA gene sequencing on fecal samples collected from HCC patients, healthy controls, and a mouse model of HCC induced by diethylnitrosamine (DEN) in combination with a choline-deficient amino acid defined high-fat diet (CDAA-HFD) (Fig. 1a). The results demonstrated a significant reduction in the abundance of the *Alistipes* genus in both HCC patients and model mice compared to healthy controls (Fig. 1b, c).

We further validated this finding using an independent cohort comprising 75 healthy controls, 40 patients with cirrhosis, and 75 patients with HCC [4]. Alistipes abundance was significantly lower in HCC patients than in healthy controls (p = 0.0139) (Table S1) and was also reduced in patients with cirrhosis, supporting its progressive depletion during chronic liver disease and HCC development.

To further dissect the functional alterations of the gut microbiota in HCC at a deeper level, we conducted metagenomic sequencing on clinical fecal samples. Analysis of alpha diversity revealed that the richness of the gut microbial community was significantly lower in HCC patients than in healthy controls (Fig. 1d). Principal coordinate analysis (PCoA) further indicated a distinct separation in microbial community structure at the genus level between the two groups (Fig. 1e). Correspondingly, the overall abundance of the *Alistipes* genus was downregulated in HCC patients (Fig. 1f). Further analysis at the species level identified that the significantly altered bacterium within this genus has not been successfully isolated or taxonomically classified (Fig. 1g). Given the current challenges in culturing this bacterium, we shifted our research focus towards its functional metabolites. The most primary fatty acid metabolite of the *Alistipes* genus is iso-15:0 [13, 15, 16], which provided a new direction for our investigation. Moreover, metagenomic analysis indicated a significant downregulation in the abundance of genes encoding key enzymes involved in the biosynthesis of monomethyl-branched-chain fatty acids (BCFAs), specifically, *BckdhA* and *TesA* in the HCC group [19] (Fig. 1h-i). Based on these findings, we directly evaluated the levels of iso-15:0 in HCC by measuring its concentration in fecal samples from both clinical patients and mouse models using gas chromatography-mass spectrometry (GC–MS). The results indicated a marked reduction in iso-15:0 levels in the HCC groups (Fig. 1j-k), suggesting that this microbial metabolite may play an important role in the pathogenesis of HCC.

### iso-15:0 suppresses the proliferation and migration of HCC cells in vitro

To investigate the biological function of iso-15:0 in HCC, we administered iso-15:0 to several HCC cell lines. iso-15:0 has been reported to induce apoptosis in various cancer models [20–23]. However, in our study, treatment with 200 μM of iso-15:0 did not elicit a significant proapoptotic effect in either PLC-PRF-5 or Huh-7 cells (Fig. S1a). This finding was further corroborated by PI staining, which confirmed the absence of substantial cell death at this concentration (Fig. S1b).

**Fig. 2:**
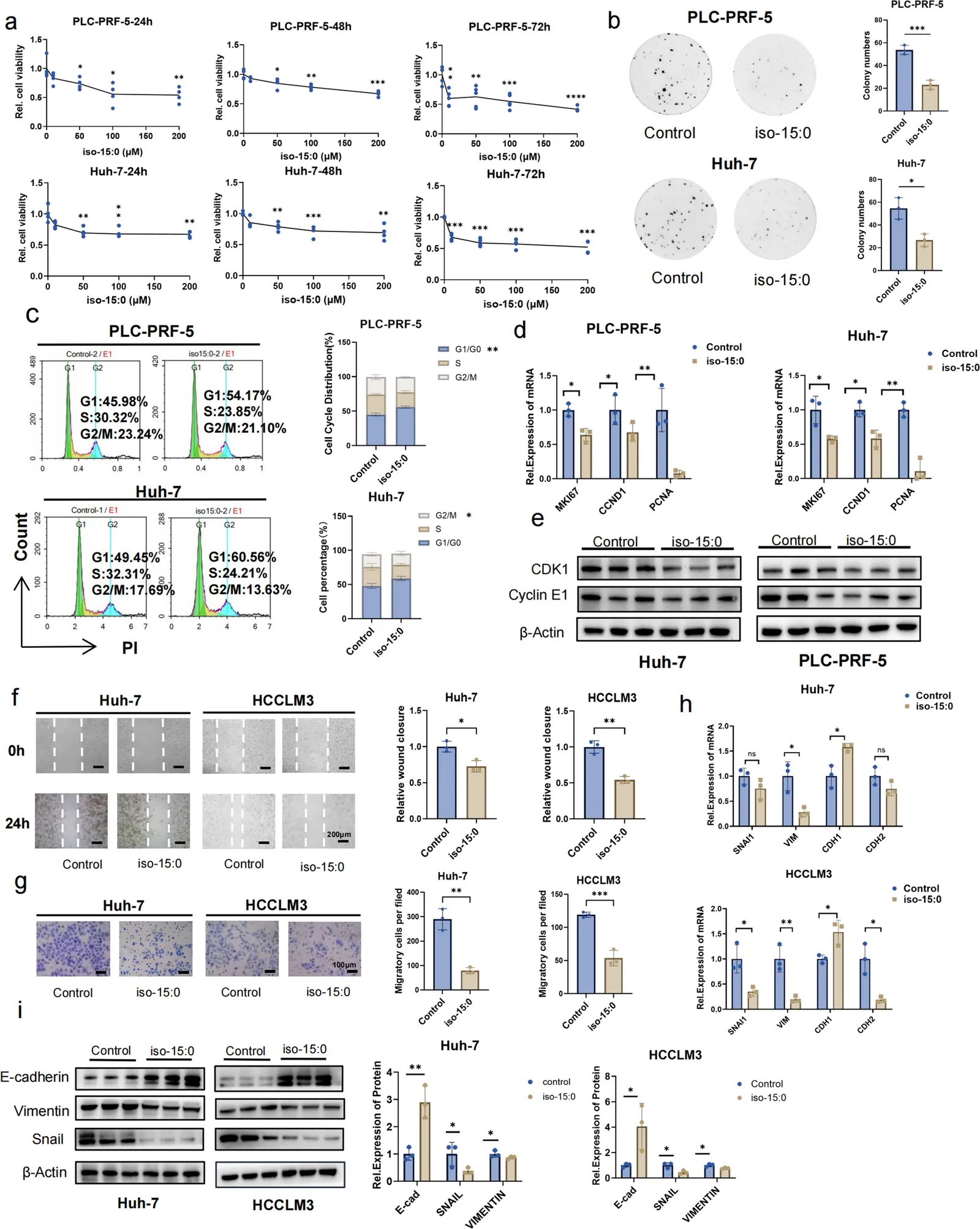
Iso-15:0 suppresses the proliferation and migration of HCC cells in vitro*.* **a** CCK-8 assays showing the viability of PLC-PRF-5 and Huh-7 cells treated with different concentrations of iso-15:0 for 24, 48, and 72 h (*n* = 4). **b** Representative images and quantitative analysis of colony formation in PLC-PRF-5 and Huh-7 cells following iso-15:0 treatment (*n* = 3). **c** Representative flow cytometry profiles and quantitative analysis of cell-cycle distribution in PLC-PRF-5 and Huh-7 cells following iso-15:0 treatment (*n* = 3). **d** RT-qPCR analysis of *MKI67*, *CCND1*, and *PCNA* mRNA expression in PLC-PRF-5 and Huh-7 cells following iso-15:0 treatment (*n* = 3). **e** Western blot analyses were conducted to measure CDK1 and Cyclin E1 protein levels in PLC-PRF-5 and Huh-7 cells after iso-15:0 exposure (*n* = 3). **f** Representative images of wound closure for Huh-7 and HCCLM3 cells treated with iso-15:0 are presented (*n* = 3). **g** Transwell assays were performed to evaluate the impact of iso-15:0 on the migration of Huh-7 and HCCLM3 cells (*n* = 3). **h** RT-qPCR assays quantified the mRNA levels of *SNAI1, CDH1, CDH2 and VIM* in cells treated with iso-15:0 (*n* = 3). **i** Western blot analyses were conducted to determine the relative protein levels of Snail1, E-cadherin, and Vimentin after iso-15:0 treatment (*n* = 3). Data are presented as mean ± SD, with significance levels indicated as *p < 0.05, **p < 0.01, ***p < 0.001, ****p < 0.0001 and ns denotes not significant

Interestingly, CCK-8 assays revealed a significant decrease in the viability of both HCC cell lines following a 48 h treatment with 50 μM iso-15:0 (Fig. 2a). Given the observation of no cell death, we hypothesized that the reduced cell viability was due to the inhibition of proliferation rather than the induction of apoptosis. To test this hypothesis, we performed a clonogenic assay, which demonstrated that 200 μM iso-15:0 markedly suppressed the colony-forming capacity of the HCC cells (Fig. 2b). Subsequent flow cytometric analysis indicated that iso-15:0 treatment induced cell cycle arrest at the G1/S phase transition (Fig. 2c). RT-qPCR analysis showed that iso-15:0 reduced the mRNA expression levels of *MKI67*, *CCND1*, and *PCNA*. Western blot analysis further demonstrated decreased protein levels of CDK1 and Cyclin E1 (Fig. 2d-e).

We next explored whether iso-15:0 affects the migratory capacity of HCC cells. We initially used highly metastatic HCCLM3 cells and further validated the findings in Huh-7 and PLC-PRF-5 cells. Wound healing and Transwell migration assays demonstrated that iso-15:0 significantly inhibited HCC cell migration, as evidenced by delayed wound closure and reduced migratory cell numbers (Fig. 2f-g, S2a-b). However, Matrigel-coated Transwell invasion assays revealed no significant impact on invasiveness (Fig. S2c). Consistent with the inhibitory effect of iso-15:0 on the migration of HCC cells, iso-15:0 treatment resulted in decreased mRNA expression levels of *Snail*, *Vimentin*, and *CDH2*, while simultaneously increasing the expression of *CDH1* in both Huh-7 and HCCLM3 cells (Fig. 2h). Similar changes in the expression of these migration-associated genes were also observed in PLC-PRF-5 cells (Fig. S2d). These alterations were further validated at the protein level, where iso-15:0 treatment led to an increase in E-cadherin expression and a decrease in Vimentin and Snail levels in Huh-7 and HCCLM3 cells (Fig. 2i).

Collectively, these findings demonstrate that iso-15:0 inhibits HCC cell proliferation and migration, highlighting its potential antitumor activity in HCC.

### iso-15:0 suppresses HCC growth and metastasis in vivo

**Fig. 3:**
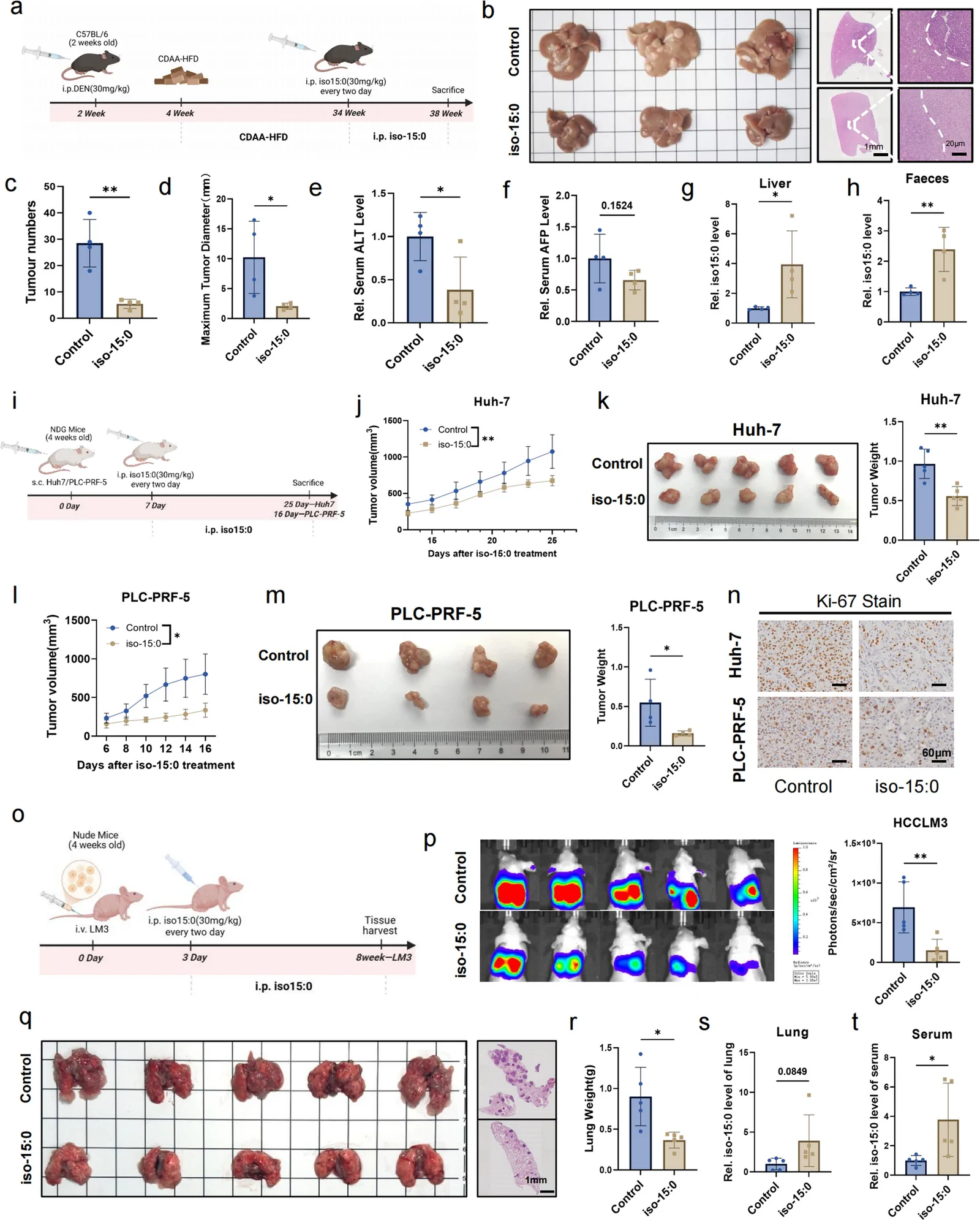
iso-15:0 suppresses tumor growth and metastasis in vivo. **a** Schematic diagram illustrating the DEN/CDAA-HFD-induced liver cancer model and the iso-15:0 administration schedule. **b** Representative gross images and H&E staining of liver tissues from control and iso-15:0-treated mice. **c**-**d** Tumor number and maximum tumor diameter in DEN/CDAA-HFD–induced HCC mice between iso-15:0-treated and control groups (*n* = 4). **e**–**f** Serum ALT and AFP levels in DEN/CDAA-HFD–induced HCC mice from control and iso-15:0-treated groups (*n* = 4). **g**-**h** iso-15:0 levels in liver tissue and fecal samples of DEN/CDAA-HFD–induced HCC mice measured by GC–MS comparing iso-15:0-treated and control groups (*n* = 4). **i** Experimental design and timeline of subcutaneous xenograft models using Huh-7 (*n* = 5) and PLC-PRF-5 (*n* = 4) cells. **j** Tumor growth curves of Huh-7 xenografts (*n* = 5). **k** Representative images and tumor weights of Huh-7 xenografts (*n* = 5). **l** Tumor growth curves of PLC-PRF-5 xenografts (*n* = 4). **m** Representative images and tumor weights of PLC-PRF-5 xenografts (*n* = 4). **n** Representative images of Ki-67 immunohistochemical staining in Huh-7 and PLC-PRF-5 subcutaneous xenograft tissues. **o** Experimental design and timeline of the HCCLM3 tail-vein lung metastasis model (*n* = 5). **p** In vivo bioluminescence imaging and quantification of metastatic burden (*n* = 5). **q** Representative gross images and H&E staining of lung tissues from control and iso-15:0-treated mice (*n* = 5). **r** Lung weights of control and iso-15:0-treated mice (*n* = 5). **s**-**t** Relative iso-15:0 levels in lung tissues (**s**) and serum (**t**), measured by GC–MS (*n* = 5). Data are presented as mean ± SD, with significance levels indicated as *p < 0.05, **p < 0.01 and ns denotes not significant

To validate the antitumor efficacy of iso-15:0 in vivo, we initially employed a mouse model of HCC induced by DEN in conjunction with a CDAA-HFD. Therapeutic intervention with iso-15:0 commenced at week 38 of the modeling period (Fig. 3a). The results indicated that, compared to the control group, iso-15:0 treatment significantly reduced both the number and maximum diameter of liver tumor nodules (Fig. 3b-d). ELISA analysis revealed a decrease in serum alanine aminotransferase (ALT), an indicator of liver injury, and alpha-fetoprotein (AFP) in the iso-15:0 treated group (Fig. 3e, f). GC–MS analysis further showed increased iso-15:0 levels in liver and fecal samples following intraperitoneal administration (Fig. 3g-h).

Furthermore, we established subcutaneous xenograft models using Huh-7 and PLC-PRF-5 cells in severely immunodeficient mice to further evaluate the antitumor effect of iso-15:0 in vivo (Fig. 3i). In the Huh-7 xenograft model, iso-15:0 treatment significantly suppressed tumor growth and reduced tumor weight compared with the control group (Fig. 3j–k). Similar inhibitory effects were observed in the PLC-PRF-5 xenograft model, as evidenced by reduced tumor volume and tumor weight (Fig. 3l–m). Consistently, immunohistochemical staining showed decreased Ki-67 expression in tumors from both Huh-7 and PLC-PRF-5 models following iso-15:0 treatment (Fig. 3n).

Next, we employed an experimental lung metastasis model established by tail-vein injection of HCCLM3 cells into nude mice. Prophylactic administration of iso-15:0 commenced on day 3 after tumor-cell inoculation (Fig. 3o). Iso-15:0 treatment significantly reduced metastatic burden and pulmonary metastatic lesions, as evidenced by decreased bioluminescence signals and histological examination of lung tissues (Fig. 3p–q). Lung weight was also markedly reduced in the iso-15:0-treated group (Fig. 3r), further supporting its inhibitory effect on HCC metastasis. Immunohistochemical staining further showed decreased Ki-67 and Vimentin expression and increased E-cadherin expression in metastatic lung lesions following iso-15:0 treatment (Fig. S3a). GC–MS analysis showed increased iso-15:0 levels in lung tissues and serum following treatment (Fig. 3s–t). Consistent results were obtained in an additional Huh-7 lung metastasis model, including reduced pulmonary metastatic lesions and lung weight, increased iso-15:0 levels in lung tissues and serum, and decreased Ki-67 expression in metastatic lesions (Fig. S3b–g).

### iso-15:0 inhibits HCC progression by suppressing the PI3K/AKT signaling pathway.

**Fig. 4:**
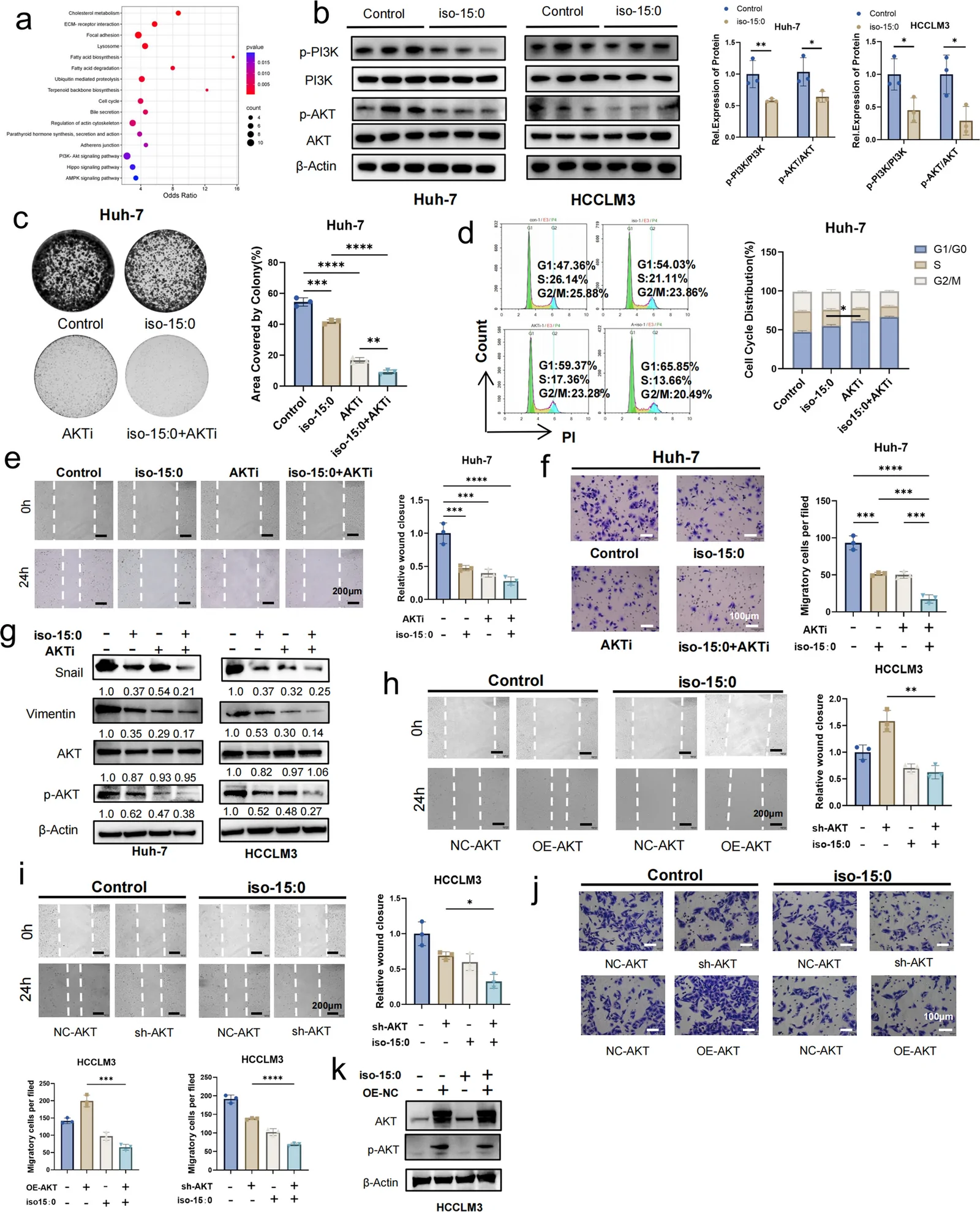
iso-15:0 inhibits the progression of HCC by suppressing the PI3K/AKT signaling pathway. **a** KEGG pathway enrichment analysis was conducted on differentially expressed genes in iso-15:0- treated cells. **b** Western blot analyses and quantitative analysis were performed to assess the levels of phosphorylated PI3K and AKT following iso-15:0 treatment (*n* = 3). **c** Colony-formation assays and quantitative analysis were conducted to evaluate the combined effects of iso-15:0 and AKT inhibition on cell proliferation (*n* = 3). **d** Cell-cycle analysis and quantitative analysis was conducted on Huh-7 cells co-treated with iso-15:0 and an AKT inhibitor (*n* = 3). **e**–**f** Wound-healing and Transwell migration assays were performed to assess cell migration following co-treatment with iso-15:0 and an AKT inhibitor, with quantitative analysis of wound closure and migrated cells, respectively (*n* = 3). **g** Western blot analyses were conducted to detect PI3K/AKT signaling activity and migration related markers post co-treatment. **h**-**i** Wound-healing assays and quantitative analysis were performed on AKT-overexpressing and AKT-knockdown cells treated with iso-15:0 (*n* = 3). **j** Transwell assays and quantitative analysis were performed to evaluate migration in AKT-overexpressing and AKT knockdown HCCLM3 cells treated with iso-15:0 (*n* = 3). **k** Western blot analyses were conducted to analyze p-AKT levels in AKT-overexpressing cells after exposure to iso-15:0. Data are presented as mean ± SD, with significance levels indicated as *p < 0.05, **p < 0.01, ***p < 0.001, ****p < 0.0001 and ns denotes not significant

To elucidate the molecular mechanism by which iso-15:0 inhibits the proliferation and metastasis of HCC cells, we conducted transcriptome sequencing analysis on Huh-7 cells treated with iso-15:0. KEGG pathway enrichment analysis identified significant alterations in pathways related to the cell cycle, cell adhesion, and PI3K/AKT signaling, highlighting PI3K/AKT signaling as a potential downstream mediator of the antitumor effects of iso-15:0 (Fig. 4a). We next examined the effects of iso-15:0 on PI3K/AKT signaling in Huh-7 and HCCLM3 cells. Western blot analysis demonstrated that iso-15:0 treatment markedly decreased the phosphorylation levels of PI3K and AKT without affecting their total protein expression, indicating that iso-15:0 specifically suppresses the activation of the PI3K/AKT pathway (Fig. 4b). Consistent with these findings, combined treatment with iso-15:0 and AKT inhibitor IV further suppressed HCC cell proliferation, colony formation, and migration, accompanied by reduced p-PI3K and p-AKT levels compared with either treatment alone (Fig. 4c–g, S4a).

To further validate the role of AKT in mediating the antitumor effects of iso-15:0, we generated HCCLM3 cells with stable AKT knockdown (shAKT) or AKT overexpression (AKT-OE) (Fig. S4b). iso-15:0 significantly inhibited migration in AKT-OE HCCLM3 cells, whereas AKT knockdown further enhanced its anti-migratory effect (Fig. 4h-i). Consistent results were also observed in the Transwell migration assay (Fig. 4j).

Additionally, we generated AKT-overexpressing Huh-7 cells (Fig. S4c), in which iso-15:0 similarly suppressed cell migration (Fig. S4d). Moreover, iso-15:0 significantly reduced p-AKT levels in AKT-overexpressing HCCLM3 cells (Fig. 4k), further supporting AKT as an important downstream mediator of the tumor-suppressive effects of iso-15:0 in HCC.

### iso-15:0 suppresses HCC progression by inhibiting Gβγ signaling

**Fig. 5:**
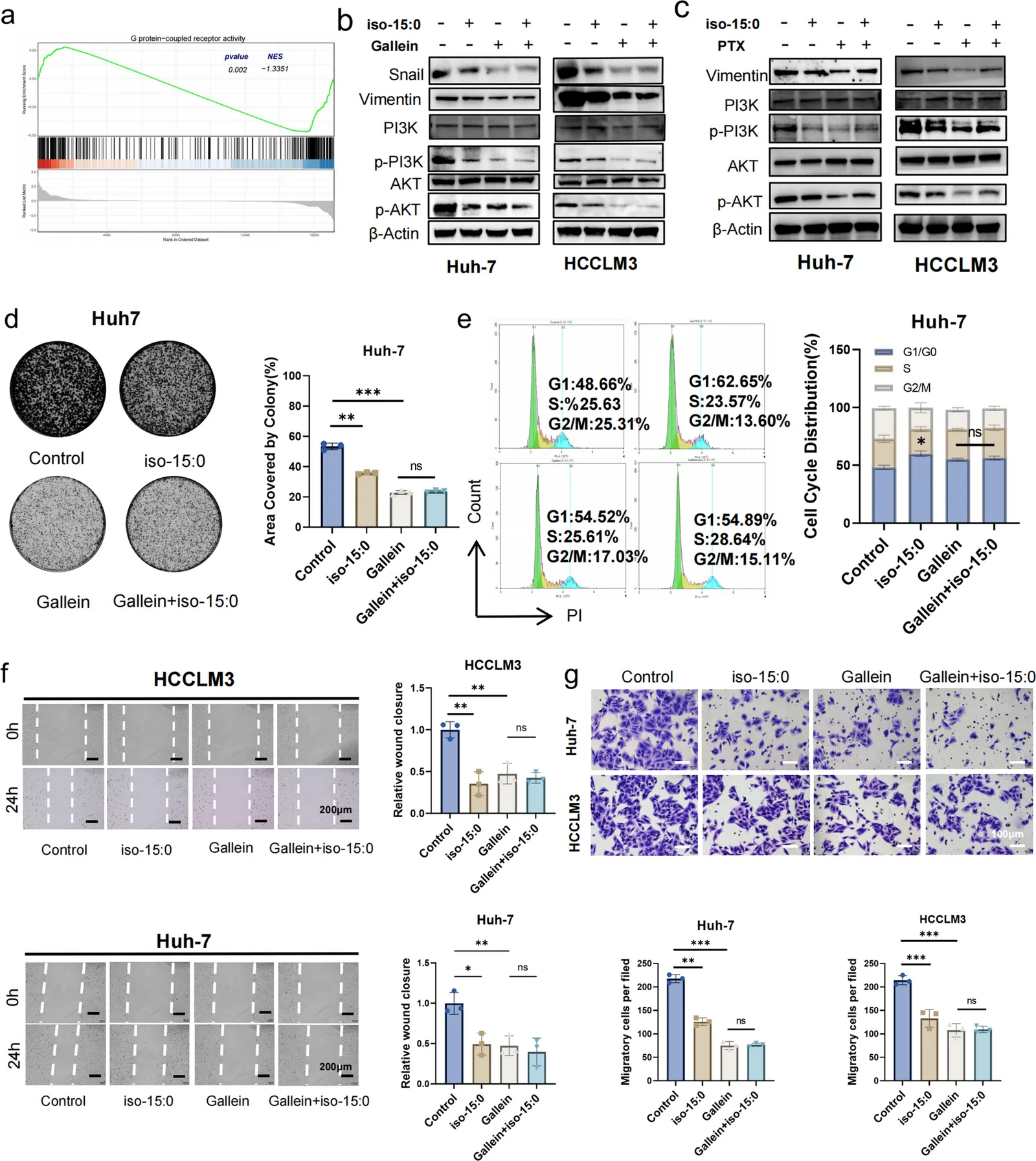
iso-15:0 suppresses the malignant progression of HCC by inhibiting Gβγ- mediated downstream signaling. **a** GSEA was conducted to identify changes in GPCR-associated signaling following iso-15:0 treatment. **b** Western blot analyses were performed to evaluate the effects of Gallein pretreatment followed by iso-15:0 on the activation of PI3K/AKT and migration-related proteins (*n* = 3). **c** Western blot analyses were performed to evaluate the effects of PTX pretreatment followed by iso-15:0 on the activation of PI3K/AKT and migration-related proteins (*n* = 3). **d** Colony formation assays and quantitative analysis were conducted to assess proliferative capacity following Gallein pathway blockade and iso-15:0 treatment (*n* = 3). **e** The distribution of the cell cycle was analyzed after Gallein pathway blockade followed by iso-15:0 treatment (*n* = 3). **f** Wound healing assays and quantitative analysis were performed to evaluate the migration abilities of Huh-7 and HCCLM3 cells after Gallein pretreatment followed by iso-15:0 treatment (*n* = 3). **g** Transwell migration assays and quantitative analysis were performed to evaluate the migration abilities of Huh-7 and HCCLM3 cells after Gallein pretreatment followed by iso-15:0 treatment (*n* = 3). Data are presented as mean ± SD, with significance levels indicated as *p < 0.05, **p < 0.01, ***p < 0.001 and ns denotes not significant

To elucidate the upstream mechanism by which iso-15:0 suppresses the PI3K/AKT signaling pathway, we initially performed GSEA. This revealed that iso-15:0 treatment significantly altered the activity of GPCR-associated signaling pathways (Fig. 5a).

Given that iso-15:0 is a branched-chain fatty acid and FFARs are lipid-sensing GPCRs, we investigated whether a receptor-mediated mechanism might contribute to its biological effects. GPR120 (FFAR4), a receptor for medium- and long-chain fatty acids, is expressed in hepatic tissues and HCC cells [24, 25]. We therefore performed molecular docking to evaluate its potential interaction with iso-15:0. iso-15:0 was predicted to occupy the GPR120 ligand-binding pocket with a binding energy of −6.7 kcal/mol (Fig. S5), suggesting a potential interaction.

Since GPCRs transduce extracellular signals through their downstream G protein subunits, particularly Gαi and Gβγ, we subsequently employed a pharmacological approach to determine which G protein component mediates the effects of iso-15:0. We utilized two specific G protein inhibitors: Gallein, which disrupts the interaction between the Gβγ subunit and PI3K, and Pertussis toxin (PTX), which inhibits Gαi subunit signaling via ADP-ribosylation. To selectively block the respective signaling pathways, cells were pre-treated with either Gallein or PTX for 8 h prior to the addition of iso-15:0, after which we assessed the phosphorylation levels of downstream targets, p-PI3K and p-AKT. Notably, when Gβγ function was fully inhibited by Gallein pre-treatment, iso-15:0 failed to further reduce the phosphorylation levels of p-PI3K and p-AKT (Fig. 5b). In contrast, when Gαi signaling was blocked by PTX pre-treatment, iso-15:0 still significantly enhanced the suppression of these phosphorylation signals (Fig. 5c). These results indicate that iso-15:0 specifically attenuates the activation of the downstream PI3K/AKT pathway by interfering with the function of the Gβγ subunit, but not the Gαi subunit.

Consistent with these findings, the expression changes of EMT-related markers, including Snail and Vimentin, followed a similar regulatory pattern (Fig. 5b). At the functional level, both cell cycle progression and clonogenic capacity, assessed after the same pre-treatment protocol, exhibited corresponding trends (Fig. 5d-e). Additionally, wound healing and Transwell migration assays demonstrated that the reduction in migratory capacity induced by the combination of Gallein and iso-15:0 was comparable to that achieved by Gallein alone (Fig. 5f-g), thereby further underscoring the pivotal role of the Gβγ subunit in mediating the inhibitory effects of iso-15:0.

### A biomimetic nanodelivery strategy enhances the therapeutic potential of iso-15:0 in HCC

**Fig. 6:**
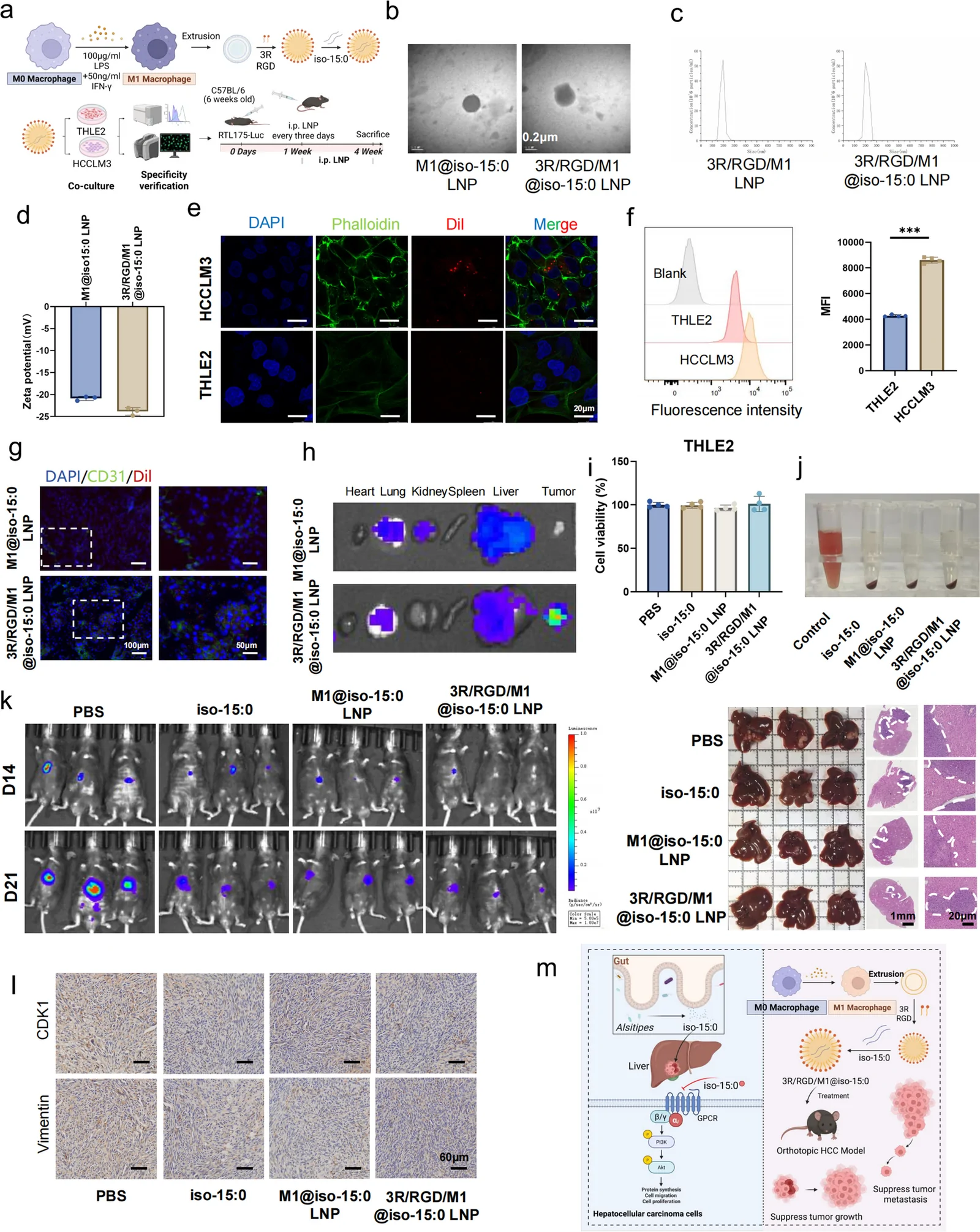
A biomimetic nanodelivery strategy enhances the therapeutic potential of iso-15:0 in HCC. **a** Schematic illustration of the preparation procedure for 3R/RGD/M1@iso-15:0 LNP. **b** Representative TEM images of M1@iso-15:0 LNP and 3R/RGD/M1@iso-15:0 LNP. **c** Hydrodynamic size distributions of 3R/RGD/M1 LNP and 3R/RGD/M1@iso-15:0 LNP measured by DLS. **d** Zeta potentials of M1@iso-15:0 LNP and 3R/RGD/M1@iso-15:0 LNP. **e** Cellular uptake of DiI-labeled 3R/RGD/M1@iso-15:0 LNP (red) in human normal hepatocyte THLE-2 and hepatocellular carcinoma HCCLM3 cells. Cells were stained for phalloidin (green) and DAPI (blue). **f** Quantitative analysis of the cellular uptake efficiency of DiI-labeled 3R/RGD/M1@iso-15:0 LNP in THLE-2 and HCCLM3 cells using flow cytometry. **g** Immunofluorescence staining of CD31 (green) and DAPI (blue) in frozen liver sections from orthotopic HCC-bearing mice following intraperitoneal injection of DiI-labeled nanoparticles. **h** Ex vivo fluorescence imaging of the heart, lung, kidney, spleen, liver, and tumor following administration of M1@iso-15:0 LNP or 3R/RGD/M1@iso-15:0 LNP. **i** Cell viability of THLE-2 cells treated with PBS, free iso-15:0, M1@iso-15:0 LNP, or 3R/RGD/M1@iso-15:0 LNP, assessed by the CCK-8 assay. **j** Representative images of the hemolysis assay following incubation with the indicated formulations. **k** Representative in vivo bioluminescence images of orthotopic HCC-bearing mice on days 14 and 21, together with gross liver images and H&E staining following treatment with PBS, free iso-15:0, M1@iso-15:0 LNP, or 3R/RGD/M1@iso-15:0 LNP. **l** Representative images of IHC staining for CDK1 and Vimentin in liver tumor tissues from the various treatment groups. **m** A graphical abstract illustrating the antitumor effects of *Alistipes*-derived iso-15:0 and its underlying mechanism, which involves Gβγ/PI3K/AKT signaling, along with the design and targeted delivery of 3R/RGD/M1@iso-15:0 LNP. Data are presented as mean ± SD, with significance levels indicated as *p < 0.05, ***p < 0.001 and ns denotes not significant

To investigate the translational potential of iso-15:0 as a therapeutic agent, we developed a biomimetic nanoplatform based on M1 macrophage membranes (3R/RGD/M1@iso-15:0 LNP), which is co-functionalized with RGD and 3R peptides [26]. M1 macrophage membranes provide intrinsic tumor tropism and immune-evasive properties [27], RGD specifically targets integrin αvβ3 [28, 29], and the liver-homing peptide 3R enhances hepatic targeting and tumor accumulation [26]. For nanoparticle preparation, M0 macrophages were polarized to the M1 phenotype and processed into membrane vesicles, followed by electroporation-mediated loading of iso-15:0 and surface conjugation of RGD and 3R peptides to generate 3R/RGD/M1@iso-15:0 LNP (Fig. 6a).

To evaluate the in vivo pharmacokinetic properties of the nanosystem, serum iso-15:0 levels were measured at different time points after administration, with correction for endogenous baseline levels (Fig. S6a). Both free iso-15:0 and 3R/RGD/M1@iso-15:0 LNP reached peak serum concentrations at 3 h; however, the nanoformulation maintained higher circulating levels throughout the observation period and increased systemic exposure by approximately 4.32-fold compared with free iso-15:0 (Fig. S6b). At 24 h after administration, free iso-15:0 was predominantly distributed in the liver, followed by the lung, kidney, spleen, and heart (Fig. S6c). These results indicate that nanoencapsulation markedly improves the in vivo pharmacokinetic profile of iso-15:0.

TEM confirmed a spherical morphology and intact membrane coating of the nanoparticles (Fig. 6b), while dynamic light scattering showed a hydrodynamic diameter of approximately 120–160 nm with a low polydispersity index and favorable zeta potential (Fig. 6c, d). DiI-labeled 3R/RGD/M1@iso-15:0 LNP showed greater uptake by HCCLM3 cells than THLE-2 cells, as determined by confocal microscopy and flow cytometry (Fig. 6e, f). Subsequent in vivo studies further confirmed the tumor-targeting advantage of the nanoplatform. In orthotopic HCC tumors, fluorescence imaging showed enhanced distribution of DiI-labeled nanoparticles beyond CD31-positive vasculature, indicating improved tumor extravasation and penetration (Fig. 6g). Consistently, ex vivo fluorescence imaging in subcutaneous tumor-bearing mice demonstrated increased tumor accumulation of 3R/RGD/M1@iso-15:0 LNP (Fig. 6h). In terms of biosafety, CCK-8 assays (Fig. 6i) revealed that the nanoplatform exhibited no significant cytotoxicity toward THLE-2 cells, while hemolysis tests (Fig. 6j) indicated excellent hemocompatibility, thereby supporting its potential for systemic administration.

Biosafety evaluation showed no obvious histopathological abnormalities in major organs following nanoparticle treatment (Fig. S7). Moreover, no significant changes in serum ALT, AST, or BUN levels or inflammatory cytokines, including IL-10, IL-1β, IL-6, and TNF-α, were observed at days 7 and 28 (Fig. S8), indicating favorable systemic biosafety.

Furthermore, we established an orthotopic HCC mouse model to evaluate the therapeutic efficacy of the nanosystem. Following the indicated treatments, in vivo bioluminescence imaging and quantitative analysis (Fig. 6k, Fig. S9) indicated that the 3R/RGD/M1@iso-15:0 LNP-treated group exhibited the greatest inhibition of tumor progression. Macroscopic examination of excised tumors, along with H&E staining (Fig. 6k), further confirmed that this group displayed the smallest tumor burden, which was accompanied by significantly reduced expression of Vimentin and CDK1 in tumor tissues (Fig. 6l).

To evaluate the contribution of dual RGD/3R modification, we compared nanoparticles modified with RGD alone, 3R alone, or both peptides. 3R/RGD/M1@iso-15:0 LNP showed greater tumor inhibition than the single-modified formulations (Fig. S10a). Consistently, fluorescence staining revealed the highest nanoparticle accumulation in tumor regions in the dual-modified group (Fig. S10b), indicating enhanced therapeutic efficacy and tumor-targeted delivery.

Collectively, these results demonstrate that the 3R/RGD/M1@iso-15:0 LNP nanosystem developed in this study effectively overcomes the targeting and delivery limitations associated with iso-15:0. By integrating the antitumor activity of iso-15:0 with a dual-targeting nanoparticle delivery strategy, this nanosystem facilitates efficient tumor accumulation and enhances the therapeutic efficacy of iso-15:0 against HCC. Mechanistically, iso-15:0 suppresses HCC progression through the inhibition of Gβγ/PI3K/AKT signaling, while the 3R/RGD/M1 LNP platform enhances targeted delivery and improves the tumor-specific action of iso-15:0 (Fig. 6m).

## Discussion

Accumulating evidence underscores the critical role of gut microbial dysbiosis in the pathogenesis of various liver diseases; however, the precise mechanisms by which gut microbes and their metabolites influence the proliferation and metastasis of HCC remain poorly defined. In this study, we integrated data from 16S rDNA sequencing, GC–MS metabolomics, and functional experiments involving HCC patients and mouse models. Our systematic investigation reveals that the *Alistipes* genus and its signature metabolite iso-15:0 play a protective role in the progression of HCC.

*Alistipes* is a relatively novel genus of Gram-negative bacteria within the *Bacteroidetes phylum*, primarily isolated from clinical specimens [9]. Accumulating evidence indicates that the *Alistipes* genus plays a protective role in liver diseases. For instance, during the progression of liver fibrosis, the abundance of *A. indistinctus*, a species within the *Alistipes* genus, is markedly downregulated [30]. Similarly, in patients with NASH and NAFLD, the abundance of *A. finegoldii* is significantly reduced compared to healthy controls [12]. These consistent observations strongly suggest a protective function of the *Alistipes* genus across the spectrum of liver diseases. However, despite these studies consistently reporting an inverse correlation between *Alistipes* abundance and disease progression, the specific metabolites responsible for mediating this protective effect remain unidentified. In our collected fecal samples from both HCC patients and mouse models, the abundance of the *Alistipes* genus was significantly lower compared to healthy controls. Consistently, other reports have confirmed a decreased abundance of *Alistipes* in the feces of early-stage HCC patients [4]. Moreover, although we observed an overall reduction in the abundance of the *Alistipes* genus within the gut microbiota of HCC patients, technical constraints prevented the identification of specific species that were significantly decreased. Notably, iso-15:0, the principal fatty acid metabolite synthesized by *Alistipes* [13, 14, 16], is biosynthesized from isoleucine *via* a bacterial enzyme-catalyzed system that is unique to bacteria and plants. Although previous studies have reported that iso-15:0 induces apoptosis and exerts anti-inflammatory effects in various cancer models, our study reveals a more nuanced functional paradigm: in HCC cells, iso-15:0 did not significantly induce apoptosis; rather, it exerted its antitumor effects by effectively inhibiting cell proliferation and migration. Moreover, in Transwell invasion assays, iso-15:0 did not significantly inhibit the matrix penetration capability of cells, indicating that its anti-metastatic effect is primarily achieved by restricting cell motility rather than impairing matrix degradation function.

The PI3K/AKT signaling pathway is aberrantly activated in numerous malignancies [31]. Its central component, AKT, facilitates cell cycle progression, inhibits apoptosis, and remodels cellular metabolism by phosphorylating various downstream substrates, thus sustaining the malignant phenotype [32]. This study reveals that iso-15:0 significantly reduces the phosphorylation levels within this pathway, thereby hindering the malignant progression of HCC. This finding establishes a vital theoretical foundation for the development of therapeutic strategies targeting HCC based on microbial metabolites.

Extensive evidence has established that fatty acids can act as ligands for GPCRs [33, 34]. Short-chain fatty acids have been well-characterized as endogenous ligands for FFAR2 and FFAR3 [35], while medium and long-chain fatty acids such as oleic acid and omega-3 polyunsaturated fatty acids primarily signal through FFAR4 [36–38]. Given that iso-15:0 is a medium-chain branched fatty acid, we hypothesized that it might act as a ligand for a specific GPCR to modulate downstream signaling. To elucidate the upstream regulatory mechanisms, transcriptomic GSEA revealed that iso-15:0 treatment significantly suppressed GPCR pathway activation. GPCRs are known to transduce diverse cellular responses *via* heterotrimeric G proteins composed of Gα, Gβ, and Gγ subunits [39]. Among downstream effectors, both Gαi and Gβγ subunits have been reported to directly or indirectly regulate the activation of the PI3K/AKT pathway [40]. According to these findings, we sought to determine which G protein subunit mediates the effect of iso-15:0 *via* pre-treating HCC cells with specific inhibitors targeting Gαi (PTX) or Gβγ (Gallein), followed by exposure to iso-15:0. The subsequent evaluation of PI3K/AKT pathway inhibition was conducted to identify the critical downstream subunit through which iso-15:0 exerts its functional effects. The results unequivocally demonstrated that the antitumor effects of iso-15:0 strictly depend on the Gβγ subunits. Specifically, iso-15:0 interferes with Gβγ-mediated PI3K/AKT signal transduction, thereby effectively inhibiting HCC proliferation and migration.

This study acknowledges several limitations. First, while our metagenomic analysis consistently revealed a significant depletion of the *Alistipes* genus in HCC, we were unable to isolate or culture the specific *Alistipes* species responsible for iso-15:0 production. As a result, direct validation of the tumor-suppressive function of this bacterium through mono-colonization or bacterial transplantation experiments was not feasible. Instead, our investigation centered on iso-15:0, its primary functional metabolite, as a surrogate to explore the biological implications of *Alistipes* depletion. Future advancements in isolating and cultivating these currently unculturable strains will be crucial for establishing a direct causal relationship between *Alistipes* colonization, iso-15:0 production, and HCC suppression. Second, although we consistently observed reductions in both *Alistipes* abundance and iso-15:0 levels within our clinical cohort, and these findings were validated using independent public microbiome datasets, the number of clinical samples included in our metabolomic analyses was relatively limited. Therefore, validation in larger, multicenter cohorts with diverse patient populations will be necessary to confirm the robustness and generalizability of these findings. Finally, while our data indicate that iso-15:0 suppresses PI3K/AKT signaling through a Gβγ-dependent mechanism, the upstream GPCR responsible for sensing iso-15:0 has yet to be reported. Identifying the direct molecular target of iso-15:0 will enhance the mechanistic framework and support future drug development.

In conclusion, our study presents a practical intervention target for HCC therapy: the restoration of *Alistipes* metabolic function or the supplementation of its active metabolite, iso-15:0. Moreover, the successful achievement of targeted delivery *via* a nanocarrier exemplifies a strategic approach to utilizing microbial metabolites in cancer treatment. In light of these findings, future research should investigate the potential of combining iso-15:0 with existing PI3K/AKT inhibitors or immune checkpoint inhibitors, with the aim of establishing novel paradigms for precision therapy in HCC.

## Materials and methods

### Human samples

This study enrolled eight patients with HCC and eight age- and gender-matched healthy controls (Table S2) from Chongqing University Cancer Hospital. The study protocol was approved by the Medical Ethics Committee of Chongqing University Cancer Hospital (Approval No.: CZLL2025-171–001). The study involving human participants was performed in compliance with the ethical requirements of the institutional review board and the principles outlined in the Declaration of Helsinki. All participants provided written informed consent before being enrolled in the study and before any biological samples were collected. The inclusion criterion for HCC patients was a confirmed diagnosis based on postoperative pathological biopsy. The exclusion criteria were: (1) administration of antibiotics, probiotics, or proton pump inhibitors within one month prior to sample collection; and (2) prior receipt of chemotherapy or radiotherapy before sample collection. Fecal samples were collected immediately after defecation, and both fecal and serum samples were stored at −80 °C.

### Animal experiments

All animal experiments were approved by the Institutional Animal Care and Use Committee of Chongqing University Cancer Hospital (Approval No.: CQCH-LAE-A0000202105) and conducted according to the 3R principles. Mice were housed under SPF conditions with controlled temperature (23 ± 2 °C), humidity (45% ± 5%), and a 12 h light/dark cycle, with free access to food and water. All mice were acclimatized for one week before experiments.

#### DEN/CDAA-HFD induced HCC model

Two-week-old male C57BL/6 J mice received a single intraperitoneal injection of diethylnitrosamine (DEN, 30 mg/kg, MCE) to initiate hepatocarcinogenesis. At four weeks of age, the diet was changed to a choline-deficient amino acid defined high-fat diet (CDAA-HFD) (ShuYu Diet, China) for continuous feeding. Intervention therapy began at week 38. Mice in the experimental group received intraperitoneal injections of iso-15:0 (30 mg/kg, MCE) every other day for four weeks. Twenty-four hours after the final administration, euthanasia was performed using CO_2_ asphyxiation. Liver tissues were collected in their entirety for gross morphological examination, tumor nodule counting, and immunohistochemical analysis.

#### Subcutaneous xenograft model

B-NDG mice were subcutaneously inoculated with 5 × 10^5^ HCC cells. When tumors reached approximately 300 mm^3^, mice were randomized and treated with iso-15:0 (30 mg/kg, i.p.) every other day until the experimental endpoint. Tumor growth was monitored by measuring tumor volume.

#### Experimental lung metastasis model

Four-week-old male BALB/c nude mice were injected *via* the tail vein with 1 × 10^5^ luciferase-expressing HCC cells. iso-15:0 treatment (30 mg/kg, *i.p*., every other day) was initiated on day 3 after inoculation. Metastatic progression was monitored by in vivo bioluminescence imaging, and lung tissues were collected at the endpoint for further analyses.

#### Orthotopic liver implantation model

Six-week-old male C57BL/6 J mice received intrahepatic injection of 5 × 10^4^ RIL-175-Luc cells in 50 μL. After comparable tumor signals were established, mice were randomized and treated with the indicated formulations at an iso-15:0-equivalent dose of 30 mg/kg (i.p.) every other day. Tumor progression was monitored by in vivo bioluminescence imaging, and liver tissues were collected at the endpoint.

### GC–MS and Fatty acid methyl ester

Lipids were extracted from approximately 50 mg of tissue using a modified Folch method with chloroform/methanol (2:1, *v/v*) and internal standards. The organic phase was collected, dried under nitrogen, and subjected to fatty acid methyl ester derivatization using methanolic NaOH (65 °C, 10 min) followed by boron trifluoride-methanol. The resulting fatty acid methyl esters were extracted with n-hexane and analyzed by GC–MS. Iso-15:0 and other fatty acids were quantified using the internal standard method based on calibrated characteristic peak areas.

### Cell culture and treatment

The human hepatocellular carcinoma cell lines Huh-7, PLC-PRF-5, and HCCLM3 utilized in this study were obtained from Procell Life Science & Technology Co., Ltd (Wuhan, China). All cell lines were cultured in Dulbecco's Modified Eagle Medium (DMEM) with high glucose (Gibco, USA), supplemented with 10% fetal bovine serum (Zeta Life, USA) and 1% penicillin–streptomycin solution (Beyotime, China). The cells were maintained in a humidified incubator at 37 °C with 5% CO_2_.

The compound iso-15:0 (MCE, USA) was freshly prepared immediately prior to each cellular treatment. Specifically, an appropriate amount of iso-15:0 was mixed with polyethylene glycol 300 (PEG300) solvent at a 1:1 (*v/v*) ratio by brief vortexing, and the resulting mixture was subsequently added directly to the cell culture system for treatment.

### Cell viability and colony formation assays

Cell viability was assessed using the CCK-8 assay. Cells were seeded in 96-well plates at 1,000 cells per well and allowed to adhere overnight, followed by treatment with the indicated concentrations of iso-15:0 for the specified durations. The CCK-8 reagent was diluted in culture medium according to the manufacturer’s instructions, added to the cells, and incubated for 2 h at 37 °C. Absorbance was measured at 450 nm using a microplate reader.

To evaluate colony-forming ability, an appropriate number of cells were plated in culture plates and treated with the indicated concentrations of iso-15:0 for 10–14 days. The culture medium was replaced every two days to ensure an adequate nutrient supply. At the conclusion of the treatment period, cells were fixed with pre-cooled methanol for 15 min and stained with a 0.5% crystal violet solution for 30 min at room temperature. The stained colonies were photographed and quantitatively analyzed using ImageJ software.

### Transwell migration and invasion assays

Cell migration and invasion abilities were assessed using Transwell chambers with an 8 μm pore size (Corning, USA). For migration assays, 1 × 10^5^ HCC cells were suspended in serum-free RPMI 1640 medium (Gibco, USA) containing 50 μM iso-15:0 and seeded into the upper chambers, while the lower chambers were filled with RPMI 1640 medium supplemented with 20% fetal bovine serum. For invasion assays, the upper chambers were pre-coated with Matrigel (1:10 dilution, 50 μL; Corning, USA) before cell seeding. After incubation for 24 h at 37 °C with 5% CO₂, cells that migrated or invaded to the lower surface of the membrane were fixed with 4% paraformaldehyde (Beyotime, China), stained with 0.1% crystal violet (Beyotime, China), and imaged using an inverted microscope.

Cell migratory capacity was further assessed using a wound healing assay. Cells were seeded into culture inserts (ibidi, Germany) placed in 24-well plates. Upon reaching approximately 90% confluence, the inserts were removed to create a uniform wound area. After gentle washing with PBS, the cells were cultured in serum-free medium containing 50 μM iso-15:0. Wound closure was monitored and photographed at 0, 24, and 48 h using a microscope.

### Cell Cycle and Apoptosis Analysis

For cell cycle analysis, hepatocellular carcinoma cells were seeded in 6-well plates and synchronized by serum starvation for 12 h. Cells were then treated with serum-free medium containing 200 μM iso-15:0 for 24 h, collected, fixed with pre-cooled ethanol, and stained with propidium iodide (PI; BD Biosciences, USA). Cell cycle distribution was analyzed by flow cytometry.

Apoptosis was detected using the Annexin V-FITC/PI staining assay. Briefly, iso-15:0-treated cells were collected and stained with Annexin V-FITC and PI according to the manufacturer's instructions (BD Biosciences, USA). The apoptotic rate was quantified by flow cytometry.

### RNA isolation and quantitative real-time PCR (RT-qPCR)

Total RNA was extracted using RNA-easy Isolation Reagent (Vazyme, China) according to the manufacturer’s instructions. RNA concentration and purity were assessed using a NanoDrop spectrophotometer, and 1 μg of qualified RNA was reverse-transcribed into cDNA using a reverse transcription kit (Selleck, China). RT-qPCR was performed using SYBR qPCR premix (Selleck, China). β-Actin served as the endogenous control, and primer sequences are listed in Table S3.

### Western blot

Protein samples were separated by SDS-PAGE and transferred onto PVDF membranes (Millipore, USA). After blocking with 5% skim milk, the membranes were incubated with primary antibodies overnight at 4 °C, followed by HRP-conjugated secondary antibodies. Protein signals were detected using ECL substrate (Thermo Fisher, USA) and quantified with ImageJ software.

### Nanoparticle preparation

M0 macrophages were polarized into M1 macrophages by stimulation with lipopolysaccharide (100 μg/mL) and interferon-γ (50 ng/mL) for 48 h. The cells were collected, lysed, and the cell membranes were extracted to generate uniform nanovesicles through sequential extrusion. iso-15:0 was loaded into the vesicles by electroporation.

The macrophage membrane-coated lipid nanoparticles (Membrane-LNP) were further functionalized with RGD tripeptide (Arg-Gly-Asp) and 3R (Arg-Arg-Arg) peptides through EDC/NHS-mediated conjugation. Briefly, carboxyl groups on the nanoparticle surface were activated and reacted with peptides to form amide bonds. After purification, the modified Membrane-LNP were resuspended in PBS for further experiments.

### Liposome morphology characterization

Samples were adsorbed onto carbon-coated copper grids for 1–2 min, rinsed with distilled water, negatively stained with 2% phosphotungstic acid (pH 7.0) for 30–60 s, and examined by transmission electron microscopy.

### Liposome size and potential measurement

Samples were diluted to a suitable concentration of approximately 10^6^ ~ 10^7^ particles/mL for analysis. The particle size distribution and average diameter were assessed using a nanoparticle analyzer at room temperature. The hydrodynamic diameter and polydispersity index of the liposomes in aqueous solution were measured *via* dynamic light scattering. Zeta potential was determined using the same instrument, with samples placed into an electrophoretic cell.

### LNP targeting validation

The targeting ability of 3R/RGD/M1@iso-15:0 LNP was evaluated in THLE-2 and HCCLM3 cells. Cells were incubated with DiI-labeled nanoparticles for 12 h, followed by flow cytometry and confocal microscopy after FITC-phalloidin and DAPI staining.

For ex vivo biodistribution analysis, subcutaneous HCC-bearing mice were intraperitoneally injected with DiI-labeled nanoparticles. At 24 h after injection, the heart, liver, spleen, lung, kidney, and tumors were collected and subjected to ex vivo fluorescence imaging.

For tumor penetration analysis, orthotopic HCC-bearing mice were intraperitoneally injected with DiI-labeled nanoparticles and sacrificed 24 h later. Frozen tumor sections were stained with CD31 antibody and DAPI, and the distribution of DiI-labeled nanoparticles relative to tumor vasculature was examined by fluorescence microscopy.

### LNP safety evaluation

THLE-2 cells were seeded in 96-well plates and treated with various concentrations of LNPs after they had adhered. Following a 48-h incubation period, cell viability was assessed using the CCK-8 assay in accordance with standard protocols. The absorbance at 450 nm was measured using a microplate reader, and survival rates were subsequently calculated.

For the hemolysis assay, fresh anticoagulated mouse blood was centrifuged to collect erythrocytes. After washing with PBS, the erythrocytes were resuspended and incubated with different nanoparticle formulations. PBS and deionized water were used as negative and positive controls, respectively. After incubation and centrifugation, the samples were photographed.

For histological evaluation, heart, liver, spleen, kidney, and lung tissues were collected from mice seven days after the initial LNP injection. Tissue sections were prepared and subjected to H&E to assess the in vivo biosafety of LNPs.

### Statistical analysis

Statistical analyses were performed using GraphPad Prism 10. Data are presented as mean ± SD. Comparisons between two groups were performed using an independent-samples *t*-test, while multiple-group comparisons were analyzed by one-way ANOVA followed by Tukey’s post hoc test. The Mann–Whitney U test was used to compare relative abundance and gene expression between HCC patients and healthy controls. *P* < 0.05 was considered statistically significant.

## Supplementary Information

Supplementary Material 1.

## Data Availability

The RNA-seq data generated in this study have been deposited in the Gene Expression Omnibus (GEO) database under accession number GSE312291. The 16S rDNA gene sequencing and metagenomic sequencing data generated in this study have been deposited in the NCBI Sequence Read Archive (SRA) under BioProject accession numbers PRJNA1372599 and PRJNA1375638, respectively. All other data supporting the findings of this study are available from the corresponding authors upon reasonable request.

## References

[1] Chan SL, Sun HC, Xu Y, Zeng H, El-Serag HB, Lee JM, et al. The Lancet Commission on addressing the global hepatocellular carcinoma burden: comprehensive strategies from prevention to treatment. Lancet. 2025;406(10504):731–78. 10.1016/s0140-6736(25)01042-6. 40744051

[2] Vogel A, Meyer T, Sapisochin G, Salem R, Saborowski A. Hepatocellular carcinoma. Lancet (London, England). 2022;400(10360):1345–62. 10.1016/s0140-6736(22)01200-4. 36084663

[3] Li C, Cai C, Wang C, Chen X, Zhang B, Huang Z. Gut microbiota-mediated gut-liver axis: a breakthrough point for understanding and treating liver cancer. Clin Mol Hepatol. 2025;31(2):350–81. 10.3350/cmh.2024.0857. PMC12016628 39659059

[4] Ren Z, Li A, Jiang J, Zhou L, Yu Z, Lu H, et al. Gut microbiome analysis as a tool towards targeted non-invasive biomarkers for early hepatocellular carcinoma. Gut. 2019;68(6):1014–23. 10.1136/gutjnl-2017-315084. PMC6580753 30045880

[5] Zhang Y, Liu W, Wong CC, Song Q, Zhang X, Zhou Q, et al. *Catenibacter iummitsuokai* promotes hepatocellular carcinogenesis by binding to hepatocytes and generating quinolinic acid. Cell Metab. 2025;37(10):1998-2013.e7. 10.1016/j.cmet.2025.09.001. 41005296

[6] Nie Q, Luo X, Wang K, Ding Y, Jia S, Zhao Q, et al. Gut symbionts alleviate MASH through a secondary bile acid biosynthetic pathway. Cell. 2024;187(11):2717-34.e33. 10.1016/j.cell.2024.03.034. 38653239

[7] Song Q, Zhang X, Liu W, Wei H, Liang W, Zhou Y, et al. *Bifidobacterium pseudolongum*-generated acetate suppresses non-alcoholic fatty liver disease-associated hepatocellular carcinoma. J Hepatol. 2023;79(6):1352–65. 10.1016/j.jhep.2023.07.005. 37459922

[8] Wu H, Chen J, Guo S, Deng J, Zhou Z, Zhang X, et al. Advances in the acting mechanism and treatment of gut microbiota in metabolic dysfunction-associated steatotic liver disease. Gut Microbes. 2025;17(1):2500099. 10.1080/19490976.2025.2500099. PMC12101596 40394806

[9] Parker BJ, Wearsch PA, Veloo ACM, Rodriguez-Palacios A. The genus *Alistipes*: gut bacteria with emerging implications to inflammation, cancer, and mental health. Front Immunol. 2020;11:906. 10.3389/fimmu.2020.00906. PMC7296073 32582143

[10] Xu YX, Liu LD, Zhu JY, Zhu SS, Ye BQ, Yang JL, et al. Alistipes indistinctus-derived hippuric acid promotes intestinal urate excretion to alleviate hyperuricemia. Cell Host Microbe. 2024;32(3):366-81.e9. 10.1016/j.chom.2024.02.001. 38412863

[11] Zheng KY, Gao B, Wang HJ, He JG, Chen HS, Hu ZL, et al. Melatonin ameliorates depressive-like behaviors in ovariectomized mice by improving tryptophan metabolism via inhibition of gut microbe *Alistipes inops*. Adv Sci. 2024;11(34):e2309473. 10.1002/advs.202309473. PMC11425877 38978348

[12] Rau M, Rehman A, Dittrich M, Groen AK, Hermanns HM, Seyfried F, et al. Fecal SCFAs and SCFA-producing bacteria in gut microbiome of human NAFLD as a putative link to systemic T-cell activation and advanced disease. United European gastroenterology journal. 2018;6(10):1496–507. 10.1177/2050640618804444. PMC6297934 30574320

[13] Song Y, Könönen E, Rautio M, Liu C, Bryk A, Eerola E, et al. *Alistipes onderdonkii* sp. nov. and *Alistipes shahii* sp. nov., of human origin. Int J Syst Evol Microbiol. 2006;56(Pt 8):1985–90. 10.1099/ijs.0.64318-0. 16902041

[14] Shkoporov AN, Chaplin AV, Khokhlova EV, Shcherbakova VA, Motuzova OV, Bozhenko VK, et al. *Alistipes inops* sp. nov. and *Coprobacter secundus* sp. nov., isolated from human faeces. Int J Syst Evol Microbiol. 2015;65(12):4580–8. 10.1099/ijsem.0.000617. 26377180

[15] Rautio M, Eerola E, Väisänen-Tunkelrott ML, Molitoris D, Lawson P, Collins MD, et al. Reclassification of *Bacteroides putredinis* (Weinberg et al., 1937) in a new genus *Alistipes* gen. nov., as *Alistipes putredinis* comb. nov., and description of *Alistipes finegoldii* sp. nov., from human sources. Syst Appl Microbiol. 1937;26(2):182–8. 10.1078/072320203322346029. 12866844

[16] Sakamoto M, Ikeyama N, Ogata Y, Suda W, Iino T, Hattori M, et al. *Alistipes communis* sp. nov., *Alistipes dispar* sp. nov. and *Alistipes onderdonkii* subsp. *vulgaris* subsp. nov., isolated from human faeces, and creation of *Alistipes onderdonkii* subsp. *onderdonkii* subsp. nov. Int J Syst Evol Microbiol. 2020;70(1):473–80. 10.1099/ijsem.0.003778. 31633480

[17] Sun LL, Liu L, Brenna JT, Wu ZH, Ma L, Bu DP. Odd- and branched-chain fatty acids in milk fat from Holstein dairy cows are influenced by physiological factors. Animal : an international journal of animal bioscience. 2022;16(6):100545. 10.1016/j.animal.2022.100545. 35636331

[18] Mao S, Liu Z, Tian Y, Li D, Gao X, Wen Y, et al. Branched-long-chain monomethyl fatty acids: are they hidden gems? J Agric Food Chem. 2023;71(48):18674–84. 10.1021/acs.jafc.3c06300. PMC10705331 37982580

[19] Wallace M, Green CR, Roberts LS, Lee YM, McCarville JL, Sanchez-Gurmaches J, et al. Enzyme promiscuity drives branched-chain fatty acid synthesis in adipose tissues. Nat Chem Biol. 2018;14(11):1021–31. 10.1038/s41589-018-0132-2. PMC6245668 30327559

[20] Cai Q, Huang H, Qian D, Chen K, Luo J, Tian Y, et al. 13-methyltetradecanoic acid exhibits antitumor activity on T-cell lymphomas in vitro and in vivo by down-regulating p-AKT and activating caspase-3. PLoS ONE. 2013;8(6):e65308. 10.1371/journal.pone.0065308. PMC3676434 23762338

[21] Lin T, Yin X, Cai Q, Fan X, Xu K, Huang L, et al. 13-Methyltetradecanoic acid induces mitochondrial-mediated apoptosis in human bladder cancer cells. Urol Oncol. 2012;30(3):339–45. 10.1016/j.urolonc.2010.04.011. 20843711

[22] Yang Z, Liu S, Chen X, Chen H, Huang M, Zheng J. Induction of apoptotic cell death and in vivo growth inhibition of human cancer cells by a saturated branched-chain fatty acid, 13-methyltetradecanoic acid. Can Res. 2000;60(3):505–9. 10676625

[23] Wongtangtintharn S, Oku H, Iwasaki H, Inafuku M, Toda T, Yanagita T. Incorporation of branched-chain fatty acid into cellular lipids and caspase-independent apoptosis in human breast cancer cell line, SKBR-3. Lipids Health Dis. 2005;4:29. 10.1186/1476-511x-4-29. PMC1315321 16305741

[24] Secor JD, Fligor SC, Tsikis ST, Yu LJ, Puder M. Free fatty acid receptors as mediators and therapeutic targets in liver disease. Front Physiol. 2021;12:656441. 10.3389/fphys.2021.656441. PMC8058363 33897464

[25] Mao C, Xiao P, Tao XN, Qin J, He QT, Zhang C, et al. Unsaturated bond recognition leads to biased signal in a fatty acid receptor. Science (New York, NY). 2023;380(6640):eadd6220. 10.1126/science.add6220. 36862765

[26] Chang T, Zheng Y, Jiang M, Jia S, Bai J, Zheng Z, et al. Peptide codes for organ-selective mRNA delivery. Nat Mater. 2025;25(1):146–59. 10.1038/s41563-025-02331-6. 40890497

[27] Zhang J, Gu B, Wu S, Liu L, Gao Y, Yao Y, et al. M1 macrophage-biomimetic targeted nanoparticles containing oxygen self-supplied enzyme for enhancing the chemotherapy. Pharmaceutics. 2023;15(9):2243. 10.3390/pharmaceutics15092243. PMC10534656 37765212

[28] Cossu J, Thoreau F, Boturyn D. Multimeric RGD-based strategies for selective drug delivery to tumor tissues. Pharmaceutics. 2023;15(2):525. 10.3390/pharmaceutics15020525. PMC9961187 36839846

[29] Yu PC, Yue CX, Dong WZ, Hao CY, Qiao YF, Liu D, et al. Cancer Immunotherapy via Disruption of Integrin αvβ3 and CD47 Costabilization on Cancer Cell Surface. Advanced science (Weinheim, Baden-Wurttemberg, Germany). 2026;13(2):e01602. 10.1002/advs.202501602. PMC12786276 41168993

[30] Shao L, Ling Z, Chen D, Liu Y, Yang F, Li L. Disorganized gut microbiome contributed to liver cirrhosis progression: a meta-omics-based study. Front Microbiol. 2018;9:3166. 10.3389/fmicb.2018.03166. PMC6315199 30631318

[31] He Y, Sun MM, Zhang GG, Yang J, Chen KS, Xu WW, et al. Targeting PI3K/Akt signal transduction for cancer therapy. Signal Transduct Target Ther. 2021;6(1):425. 10.1038/s41392-021-00828-5. PMC8677728 34916492

[32] Song M, Bode AM, Dong Z, Lee MH. AKT as a Therapeutic Target for Cancer. Can Res. 2019;79(6):1019–31. 10.1158/0008-5472.Can-18-2738. 30808672

[33] Liu H, Zhang Q, He X, Jiang M, Wang S, Yan X, et al. Structural insights into ligand recognition and activation of the medium-chain fatty acid-sensing receptor GPR84. Nat Commun. 2023;14(1):3271. 10.1038/s41467-023-38985-6. PMC10241960 37277332

[34] Lin H, Ma C, Cai K, Guo L, Wang X, Lv L, et al. Metabolic signaling of ceramides through the FPR2 receptor inhibits adipocyte thermogenesis. Science (New York, NY). 2025;388(6746):eado4188. 10.1126/science.ado4188. 40080544

[35] de Vos WM, Tilg H, Van Hul M, Cani PD. Gut microbiome and health: mechanistic insights. Gut. 2022;71(5):1020–32. 10.1136/gutjnl-2021-326789. PMC8995832 35105664

[36] Hilgendorf KI, Johnson CT, Mezger A, Rice SL, Norris AM, Demeter J, et al. Omega-3 fatty acids activate ciliary FFAR4 to control adipogenesis. Cell. 2019;179(6):1289-305.e21. 10.1016/j.cell.2019.11.005. PMC7332222 31761534

[37] Oh DY, Walenta E. Omega-3 fatty acids and FFAR4. Front Endocrinol. 2014;5:115. 10.3389/fendo.2014.00115. PMC4100060 25076939

[38] Kimura I, Ichimura A, Ohue-Kitano R, Igarashi M. Free Fatty Acid Receptors in Health and Disease. Physiol Rev. 2020;100(1):171–210. 10.1152/physrev.00041.2018. 31487233

[39] Li L, An Z, Lin C, Xu Q, Tang C. An update on regulation and function of G protein-coupled receptors in cancer: A promising strategy for cancer therapy. Biochim Biophys Acta. 2025;1880(2):189266. 10.1016/j.bbcan.2025.189266. 39864470

[40] Zhang M, Chen T, Lu X, Lan X, Chen Z, Lu S. G protein-coupled receptors (GPCRs): advances in structures, mechanisms, and drug discovery. Signal Transduct Target Ther. 2024;9(1):88. 10.1038/s41392-024-01803-6. PMC11004190 38594257

