# Supplementary material for: Alistipes-derived monomethyl branched-chain fatty acid iso-15:0 suppresses hepatocellular carcinoma progression by inhibiting Gβγ/PI3K/AKT signaling
Source: Mol Biomed. 2026 Oct 14;7:211. doi: 10.1186/s43556-026-00622-1 (PMC13669273; doi:10.1186/s43556-026-00622-1)
Supplement: Supplementary file 1 [file 43556_2026_622_MOESM1_ESM.docx]

***Alistipes*-Derived Monomethyl Branched-Chain Fatty Acid iso-15:0 Suppresses Hepatocellular Carcinoma Progression by Inhibiting Gβγ/PI3K/AKT Signaling**

Juan He1#, Yu Chen1,2,3#, Liwen Kuang1#, Yang Xiao1#, Jiongming Chen1, Xudong Liu1, Jiangang Zhang1,2,3, Ziyi Li1, Dongqin Zhou1, Miaoyu Song1, Yi Yi1, Danyang Li1, Haili Shang1, Nan Zhang1, Ran Ren1, Yu Zhou1,2,3, Juan Lei1,2,3, Huakan Zhao1,2,3*, Yongsheng Li1,2,3*

^1^ School of Medicine, Department of Medical Oncology of Chongqing University Cancer Hospital, Chongqing University, Chongqing 400030, China

^2^ Chongqing Key Laboratory for the Mechanism and Intervention of Cancer Metastasis, Chongqing 400030, China

^3^ Key Laboratory of Chongqing Education Commission of China for Cancer Immunometabolism Translational Research, Chongqing, 400030, China

**Figure S1. iso-15:0 treatment does not induce apoptosis or cell death in HCC cells.**

(a) Apoptosis in HCC cells treated with iso-15:0 was detected using flow cytometry, employing Annexin V-FITC/PI dual staining (n=3).

(b) The live/dead ratio of HCC cells subjected to iso-15:0 treatment was assessed through flow cytometry utilizing propidium iodide (PI) staining (n=3).

Data are presented as mean ± SD, ns denotes not significant.

**Figure S2. iso-15:0 inhibits the migration of HCC cells but does not suppress invasion.**

(a) Wound-healing assays were conducted to assess the migratory capacity of PLC-PRF-5 cells following treatment with iso-15:0 (n=3).

(b) Cell migration was evaluated using a Transwell assay (without Matrigel coating) for PLC-PRF-5 cells exposed to iso-15:0 (n=3).

(c) Cell invasion was assessed using a Matrigel-coated Transwell assay in Huh-7 and HCCLM3 cells treated with iso-15:0 (n=3).

(d) RT-qPCR analysis was conducted to measure the mRNA expression levels of *SNAI1*, *CDH1*, *CHD2*, and *VIM* in PLC-PRF-5 cells after iso-15:0 treatment (n=3).

Data are presented as mean ± SD, with significance levels indicated as *p < 0.05, **p < 0.01, ns denotes not significant.

**Figure S3. iso-15:0 suppresses tumor growth and metastasis *in vivo*.**

(a) Representative immunohistochemical staining of Ki-67, Vimentin, and E-cadherin in HCCLM3 metastatic lung lesions from the indicated treatment groups.

(b) A schematic diagram illustrating the experimental design and timeline for establishing the experimental lung metastasis model through tail vein injection of Huh-7 cells into nude mice.

(c) Gross morphology and representative H&E staining images of lung tissues from mice across different treatment groups.

(d) Quantification of lung tissue weight from mice in each group (n = 5).

(e-f) iso-15:0 levels in lung tissue and serum (n = 5).

(g) Representative Ki-67 immunohistochemical staining of metastatic lung lesions in the Huh-7 lung metastasis model.

Data are presented as mean ± SD, with significance levels indicated as *p < 0.05.

**Figure S4. iso-15:0 inhibits the progression of HCC by suppressing the PI3K/AKT signaling pathway.**

(a) Transwell migration assays were conducted to evaluate the migratory ability of HCCLM3 cells treated with a combination of iso-15:0 and an AKT inhibitor (n=3).

(b) Western blot and RT-qPCR analyses were performed to assess the efficiency of AKT knockdown and overexpression at both the protein and mRNA levels in HCCLM3 cells.

(c) Western blot analyses were executed to confirm AKT overexpression at the protein level in Huh-7 cells.

(d) Transwell migration assays were utilized to investigate the migratory ability of AKT-overexpressing Huh-7 cells following iso-15:0 treatment (n=3).

Data are presented as mean ± SD, with significance levels indicated as *p < 0.05, **p < 0.01, ***p < 0.001, and ****p<0.0001.

**Figure S5. Predicted interaction between iso-15:0 and GPR120 based on molecular docking analysis.**

Molecular docking analysis was performed to predict the potential interaction between iso-15:0 and the free fatty acid receptor GPR120 (FFAR4). The three-dimensional binding mode of iso-15:0 within the predicted ligand-binding pocket of GPR120 is shown. The enlarged view illustrates the spatial interactions between iso-15:0 and surrounding amino acid residues of GPR120. Molecular docking analysis predicted a favorable binding affinity between iso-15:0 and GPR120, with a binding energy of −6.7 kcal/mol.

**Figure S6. Pharmacokinetic profile and tissue distribution of free iso-15:0 and 3R/RGD/M1@iso-15:0 LNP.**

(a) Serum concentration-time profiles of iso-15:0 following intraperitoneal administration of free iso-15:0 and 3R/RGD/M1@iso-15:0 LNP in mice. The concentrations of iso-15:0 in serum were measured at indicated time points (0, 3, 6, 12, and 24 h) (n=3).

(b) Pharmacokinetic analysis of systemic iso-15:0 exposure following administration of free iso-15:0 or 3R/RGD/M1@iso-15:0 LNP.

(c) Tissue distribution analysis of iso-15:0 in major organs, including heart, liver, spleen, lung, and kidney.

Data are presented as mean ± SD, with significance levels indicated as *p < 0.05.

**Figure S7. Biocompatibility and toxicity assessment of nanoparticles *in vivo*.**

H&E staining of major organs (heart, liver, spleen, lung, and kidney) from nanoparticle-treated mice.

**Figure S8. Time-dependent *in vivo* biosafety evaluation of 3R/RGD/M1@iso-15:0 LNP administration in mice.**

(a) Serum levels of inflammatory cytokines, including IL-10, IL-1β, IL-6, and TNF-α, and biochemical indicators of liver and kidney function, including ALT, AST, and BUN, were measured 7 days after administration of PBS, 3R/RGD/M1 LNP, or 3R/RGD/M1@iso-15:0 LNP.

(b) Serum levels of inflammatory cytokines, including IL-10, IL-1β, IL-6, and TNF-α, and biochemical indicators of liver and kidney function, including ALT, AST, and BUN, were measured 28 days after administration of PBS, 3R/RGD/M1 LNP, or 3R/RGD/M1@iso-15:0 LNP.

Data are presented as mean ± SD, ns denotes not significant.

**Figure S9. Quantitative analysis of *in vivo* bioluminescence intensity in orthotopic HCC-bearing mice after treatment with different formulations.**

Quantitative analysis of *in vivo* bioluminescence intensity in orthotopic HCC-bearing mice on days 14 and 21 following treatment with PBS, free iso-15:0, M1@iso-15:0 LNP, or 3R/RGD/M1@iso-15:0 LNP.

**Figure S10. Therapeutic efficacy and tumor-targeting analysis of different LNP formulations.**

(a) Representative images of liver tissues and H&E staining from orthotopic HCC-bearing mice treated with PBS, iso-15:0, M1@iso-15:0 LNP, 3R/M1@iso-15:0 LNP, RGD/M1@iso-15:0 LNP, or 3R/RGD/M1@iso-15:0 LNP.

(b) Immunofluorescence staining of orthotopic tumor sections after administration of DiI-labeled LNPs. Tumor sections were stained with DAPI (blue) and CD31 (green), and DiI fluorescence (red) was detected to visualize nanoparticle distribution.

| Groups | Number of cases | Rel. abundance (%) | | Significance (vs. HCC) | | |
| --- | --- | --- | --- | --- | --- | --- |
|  |  | Mean | Median | P | BH | Q |
| Healthy | 75 | 1.9992 | 1.49 | 0.0139 | 0.2113 | 0.211 |
| Cirrhosis | 40 | 1.1165 | 0.215 | 0.176715 | 0.44678 | 0.28793 |
| HCC | 75 | 1.17 | 0.52 | / | / | / |

Table S1. The relative abundance of the *Alistipes* genus in fecal samples from HCC patients, Cirrhosis patients and healthy controls was assessed using 16S rRNA gene sequencing. Data from **Zhigang Ren *et al*. Gut 2019;68:1014-1023**.

Table S2. Clinical pathological information of HCC patients

|  | gender | age | postoperative pathological staging |
| --- | --- | --- | --- |
| HCC1 | Male | 46 | Hepatocellular carcinoma Ⅳstage |
| HCC2 | Male | 58 | Hepatocellular carcinoma Ⅳstage |
| HCC3 | Male | 46 | Hepatocellular carcinoma Ⅳstage |
| HCC4 | Male | 47 | Hepatocellular carcinoma Ⅳstage |
| HCC5 | Male | 35 | Hepatocellular carcinoma Ⅳstage |
| HCC6 | Female | 58 | Hepatocellular carcinoma Ⅲstage |
| HCC7 | Female | 52 | Hepatocellular carcinoma Ⅳstage |
| HCC8 | Female | 35 | Hepatocellular carcinoma Ⅳstage |

|  | Name | Sequence |
| --- | --- | --- |
| Forward Sequence | *AKT* | TGGACTACCTGCACTCGGAGAA |
| Reverse Sequence | *AKT* | GTGCCGCAAAAGGTCTTCATGG |
| Forward Sequence | *CDK1* | GGAAACCAGGAAGCCTAGCATC |
| Reverse Sequence | *CDK1* | GGATGATTCAGTGCCATTTTGCC |
| Forward Sequence | *MKI67* | GAAAGAGTGGCAACCTGCCTTC |
| Reverse Sequence | *MKI67* | GCACCAAGTTTTACTACATCTGCC |
| Forward Sequence | *PCNA* | CAAGTAATGTCGATAAAGAGGAGG |
| Reverse Sequence | *PCNA* | GTGTCACCGTTGAAGAGAGTGG |
| Forward Sequence | *SNAI1* | TGCCCTCAAGATGCACATCCGA |
| Reverse Sequence | *SNAI1* | GGGACAGGAGAAGGGCTTCTC |
| Forward Sequence | *VIM* | AGGCAAAGCAGGAGTCCACTGA |
| Reverse Sequence | *VIM* | ATCTGGCGTTCCAGGGACTCAT |
| Forward Sequence | *CDH1* | GCCTCCTGAAAAGAGAGTGGAAG |
| Reverse Sequence | *CDH1* | TGGCAGTGTCTCTCCAAATCCG |
| Forward Sequence | *CDH2* | CCTCCAGAGTTTACTGCCATGAC |
| Reverse Sequence | *CDH2* | GTAGGATCTCCGCCACTGATTC |
| Forward Sequence | *CCND1* | TCTACACCGACAACTCCATCCG |
| Reverse Sequence | *CCND1* | TCTGGCATTTTGGAGAGGAAGTG |
| Forward Sequence | *Beta Actin* | CACCATTGGCAATGAGCGGTTC |
| Reverse Sequence | *Beta Actin* | AGGTCTTTGCGGATGTCCACGT |

Table S3. List of primers used in this study
